# Aldehyde dehydrogenase 2 and NOD-like receptor thermal protein domain associated protein 3 inflammasome in atherosclerotic cardiovascular diseases: A systematic review of the current evidence

**DOI:** 10.3389/fcvm.2023.1062502

**Published:** 2023-02-23

**Authors:** Xue-yun Shi, Xiao-lin Yue, You-shun Xu, Mei Jiang, Rui-jian Li

**Affiliations:** ^1^Qilu Medical College, Shandong University, Jinan, China; ^2^Department of Emergency, Qilu Hospital, Shandong University, Jinan, China

**Keywords:** atherosclerosis, NLRP3 inflammasome, oxidative stress, inflammatory response, mitochondrial damage, ALDH2, Nrf2, CD36

## Abstract

Inflammation and dyslipidemia underlie the pathological basis of atherosclerosis (AS). Clinical studies have confirmed that there is still residual risk of atherosclerotic cardiovascular diseases (ASCVD) even after intense reduction of LDL. Some of this residual risk can be explained by inflammation as anti-inflammatory therapy is effective in improving outcomes in subjects treated with LDL-lowering agents. NOD-like receptor thermal protein domain associated protein 3 (NLRP3) inflammasome activation is closely related to early-stage inflammation in AS. Aldehyde dehydrogenase 2 (ALDH2) is an important enzyme of toxic aldehyde metabolism located in mitochondria and works in the metabolism of toxic aldehydes such as 4-HNE and MDA. Despite studies confirming that ALDH2 can negatively regulate NLRP3 inflammasome and delay the development of atherosclerosis, the mechanisms involved are still poorly understood. Reactive Oxygen Species (ROS) is a common downstream pathway activated for NLRP3 inflammasome. ALDH2 can reduce the multiple sources of ROS, such as oxidative stress, inflammation, and mitochondrial damage, thereby reducing the activation of NLRP3 inflammasome. Further, according to the downstream of ALDH2 and the upstream of NLRP3, the molecules and related mechanisms of ALDH2 on NLRP3 inflammasome are comprehensively expounded as possible. The potential mechanism may provide potential inroads for treating ASCVD.

## 1. Introduction

Atherosclerosis (AS) is important in the development of cardiovascular and cerebrovascular diseases. Atherosclerotic cardiovascular disease (ASCVD) is the disease with the highest incidence rate and mortality in human beings, accounting for about 1/3 of global deaths (1). Therefore, exploring its pathological mechanisms and seeking effective treatment is of great social significance.

Inflammation and lipid metabolism disorder are the pathophysiological basis of atherosclerosis. A key feature of ASCVD is the production of foam cells, characterized by the utilization and aggregation of oxidized low-density lipoprotein (ox-LDL) by sub-endothelial macrophages known as foam cells (FC). This accretion of FC contributes to lipid storage as plaque and sustains its growth. Macrophages secrete pro-inflammatory cytokines, chemokines and produce reactive oxygen species (ROS) maintaining a local inflammatory response. Several research results have shown that NLRP3 (nucleus oligomerization domain like receptor family, pyrin domain containing 3) inflammasome is closely related to macrophage function and phenotypic transformation. Activation the of NLRP3 related pathways is closely related to the development and stability of plaques (2–5).

The NLRP3 inflammasome is a multimeric cytosolic protein complex composed of three proteins, including receptor protein (NLRP3), connexin (ASC) and effector protein (pro-caspase-1). Its activation process is divided into a priming and an activation stage. At the priming stage, pattern recognition receptors (PRRs) on the cell surface, such as toll like receptors (TLRs) and interleukin-1 receptors (IL-1R), activate nuclear factors after recognizing extracellular pathology related molecular patterns (PAMPs) and danger related molecular patterns (DAMPs). Nuclear factors-κB(NF-κB) signal pathway is then activated, promoting NLRP3 inflammasome related gene NLRP3, apoptosis related microparticle protein (ASC), caspase-1 and interleukin-1β (IL-1β) transcription and translation. The activation phase is the key to NLRP3 activation.

Activation of NLRP3 inflammasome produces IL-1β and cause cell death. The dead cells release a series of inflammatory factors such as ATP, heat shock protein and pro-inflammatory interleukins such as IL-1β, IL-18. In addition, the pyroptotic cells can also promote the enlargement plaque’s necrotic core and accelerate its progress. Our previous study found that the up-regulation of ALDH2 activity can significantly reduce oxidative stress and inflammatory response in human and ApoE^−/−^ mouse as plaques, and then affect the progress of AS (6).

Clinical studies have shown that there is a significant negative correlation between the severity of atherosclerotic plaque and the activity of Aldehyde dehydrogenase 2 (ALDH2) in patients with coronary heart disease (7). It was found that ALDH2 activity was strongly associated to the formation, stability, and inflammation of plaque. Alda-1, a specific agonist of ALDH2, can reduce the plaque area of ApoE^−/−^ mouse (8). ALDH2 activation can simultaneously inhibit the priming and activation of NLRP3 inflammasome, and then inhibit pyroptosis. Further exploration of its regulatory mechanism can provide further insight for potential treatment strategies targeting NLRP3 inflammasome to inhibit the development of atherosclerosis.

## 2. Inflammation and residual risk of atherosclerotic cardiovascular diseases (ASCVD)

**Figure 1:**
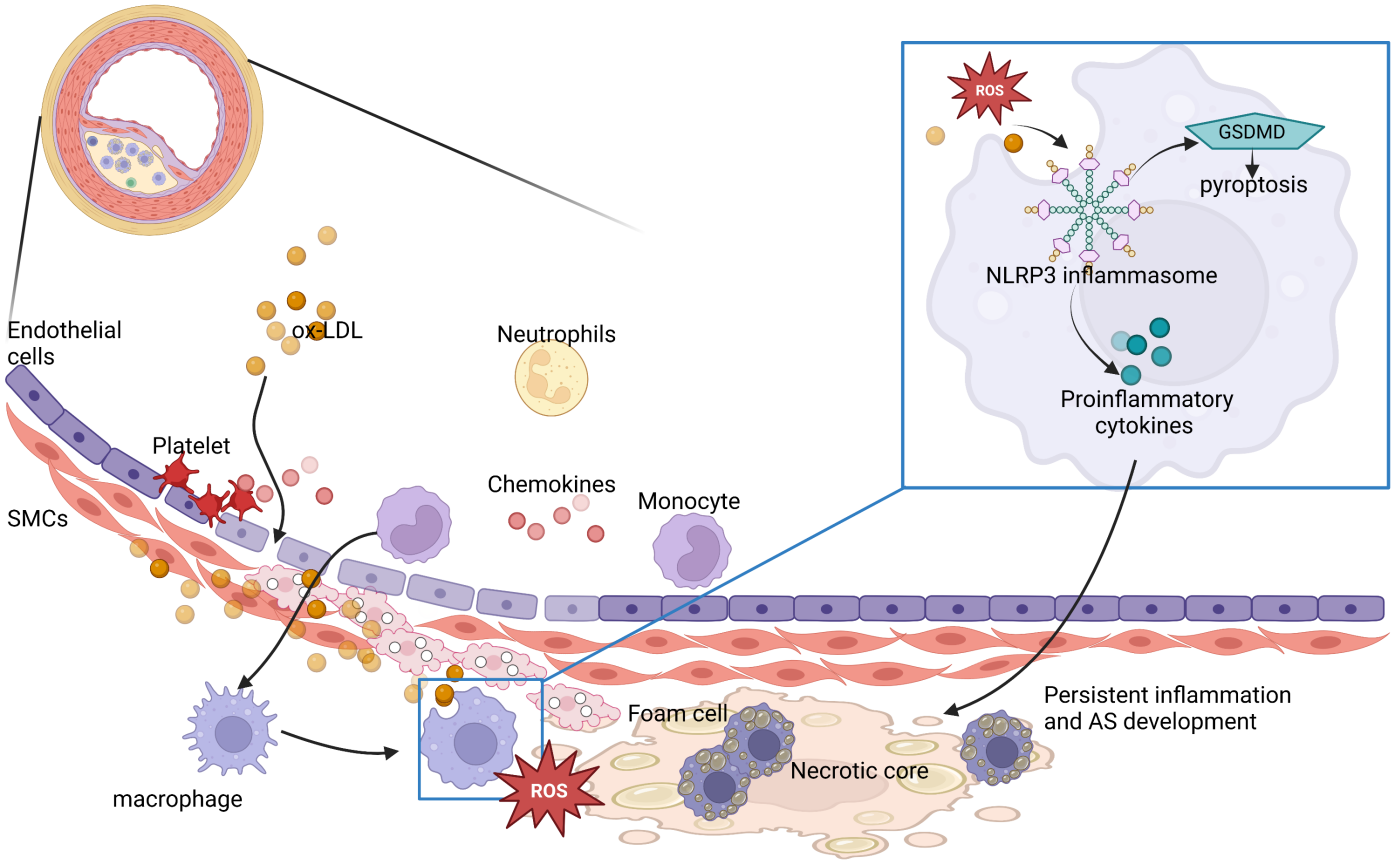
NLRP3 inflammasomes participate in and play an amplifying role in the development of atherosclerotic inflammation. The process is represented by ox-LDL in the Figure 1. ROS, reactive oxygen species; AS, atherosclerosis.

There is now overwhelming experimental and clinical evidence that atherosclerosis is a chronic inflammatory disease (9). The most common atherosclerosis is a lipid-driven inflammatory disease of the interior arterial wall and endothelial surface in which the balance of pro or anti-inflammatory mechanisms dictates the final clinical outcome (10). Lipid deposition in the intima begins before atherosclerosis. Low-density lipoprotein (LDL) accumulation initiates vascular inflammation. ROS modifies LDL to oxidized LDL (ox-LDL) and collects in the internal wall of the vasculature promoting atherosclerotic plaque development (11). In addition, oxidized phospholipids trigger inflammation by binding to Toll-like receptors (TLRs) that can cause pro-inflammatory signaling (9). In the intima, innate immune cells are stimulated to remove deposited lipoproteins and secrete pro-inflammatory cytokines. Monocytes transform into macrophages and incorporate the atherogenic lipoproteins by scavenger receptors, such as CD36 (12). After ox-LDL enters cells, the inflammatory response is enhanced by the activation of the NLRP3 inflammasome in macrophages (10). Then macrophages secrete proinflammatory cytokines, chemokines and produce ROS to maintain local inflammatory response and ingest and accumulate ox-LDL to produce foam cells. The accretion of foam cells contributes to plaque lipid storage and its sustained growth. However, too much inflammation gradually leads to macrophage death, further aggravating inflammatory response and induce chronic inflammation in plaques. At the same time, macrophage death by apoptosis and necrosis promotes formation of a soft lipid-rich core inside the plaque. In addition, macrophages interact with vascular smooth muscle cells, amplify the inflammatory cycle by producing excessive pro-inflammatory cytokines and extracellular matrix components, and further promote the retention of lipoproteins (9, 11). This persistent inflammation will drive programmed necrosis of macrophages, causing buildup of debris and necrotic cells, thereby promoting the formation of necrotic cores in atherosclerotic plaques (Figure 1).

An important clinical result of cholesterol loading in AS is the formation of intracellular cholesterol microcrystals that activate the NLRP3 inflammasome, which cleaves pro-IL-1β into its biologically active form IL-1β to enhance the expression of pro-inflammatory cytokines (9, 13). Takashi et al. (14) have confirmed that NLRP3 inflammasome plays a decisive role in atherosclerosis, and the activation of NLRP3 related pathways is closely related to the development and stability of plaque. Because ALDH2 activity was closely related to the formation, stability, and inflammation of plaque, ALDH2 activation can inhibit the activation of NLRP3 inflammasome. Thus, atherosclerosis is an inflammatory driven process and the NLRP3 as an early triggering factor and the core of vicious circle. ALDH2 is one of the most critical regulatory factors.

During the development of AS, ALDH2 activity on NLRP3 can promote the formation and progress of plaque and affect its stability and mediate its rupture (10, 15, 16). Accordingly, the residual risks of ASCVD include residual cholesterol risk and residual inflammation risk. Their main biological indicators are low-density lipoprotein cholesterol (LDL-C) and high-sensitivity C-reactive protein (hs-CRP).

Based on the established relation between increased blood cholesterol, particularly LDL-C, and ASCVD, current treatment procedures aim on reducing LDL-C to lower risk of ASCVD (17, 18). Several large-scale clinical trials have shown that targeting the NLRP3 inflammasome and the IL-1β pathway represents a new opportunity to reduce remaining risk in patients with ASCVD (13).

It can be seen from the above that the priming and activation of NLRP3 inflammatory bodies play a clue role in the typical chronic inflammation of AS. The activation of ALDH2 can inhibit the priming and activation of NLRP3 inflammatory body, thus slowing down the inflammatory process and reducing the residual risk. Therefore, study of the regulatory mechanism of ALDH2 on NLRP3 will provide new ideas for the treatment of atherosclerosis.

## 3. The activation and regulation of NLRP3 inflammasome

NLRP3 is an intracellular immune receptor closely related to lipid metabolism disorder and inflammatory response. The inflammasome formation is in response to harmful stimuli from pathogens and damaged or dead cells. These are known as pathogen-associated molecular patterns (PAMPs) or damage/danger-associated molecular patterns (DAMPs) (19). NLRP3 identifies a broad assortment of stimuli and is associated in the origination and development of sterile inflammatory diseases (14).

The NLRP3 inflammasome consists of the sensor NLRP3, adaptor ASC, and pro-caspase-1enzyme. The C-terminal contains a leucine-rich repeat domain (LRR), a central nucleotide-binding and oligomerization domain (NACHT or NOD), and an N-terminal pyrin domain (PYD). ASC contains PYD and CARD. Pro-caspase-1 is made up of a CARD and catalytic domains termed p20/p10 subunits.

NLRP3 is activated by changes to its conformation. After, it recruits ASC through a PYD-PYD interaction. Pro-caspase-1 is added through a CARD–CARD interaction. Assembly of NLRP3, ASC, and pro-caspase-1 causes cleavage of pro-caspase-1 leading to create and release catalytically active subunits p20/10 (14).

### 3.1. The NLRP3 inflammasome activation process is divided into two steps: Priming and activation

The level of inflammasome associated protein increased in the priming stage. Pattern recognition receptors (PRRS) on the cell surface, such as TLRs and IL-1R, activate the NF-κB signal pathway after recognizing extracellular PAMPs and DAMPs to promote transcription and translation of NLRP3 inflammasome related gene NLRP3, ASC, caspase-1, and IL-1β.

The activation phase is the key to NLRP3 activation. It promotes tissue damage following acute organ inflammatory injury (20). Mature caspase-1 cleaves gasdermin protein (GSDMD). Mature GSDMD protein is embedded into the cell membrane to form 10–14 nm pores, which mediates pyroptosis (21, 22). Pyroptotic cells release a series of inflammatory factors such as ATP, heat shock protein (HSP) and pro-inflammatory factors such as IL-1β and IL-18. IL-1β activates the inflammatory cascade and expand the inflammatory response (19, 22), while IL-1β combines with IL-1R of macrophages to promote the initiation of NLRP3 which in turn expands the scope of inflammation. In addition, the pyroptotic cells also promote the growth of the plaque’s necrotic core and accelerate the its progression (23). All these accelerate the development of AS.

### 3.2. NLRP3 can be activated by many signals

#### 3.2.1. Potassium ion (K^+^) outflow and calcium ion (Ca^2 +^) inflow

K^+^ efflux is one of the important upstream signaling pathways in NLRP3 activation. It has been found that Nigerian bacteriocin and ATP can promote the activation of NLRP3 inflammasome by promoting potassium ion outflow. Research shows that K^+^ outflow is accompanied by the opening of intracellular Ca^2+^ channel and the release of endoplasmic reticulum Ca^2+^, which jointly promote the activation of NLRP3 (24). NEK7 (NIMA-related kinase 7)is a key regulator of NLRP3 activation (25). It acts downstream of K^+^ efflux and promotes NLRP3 oligomerization, ASC spot formation and caspase-1 activation.

#### 3.2.2. Instability and rupture of lysosomes

Phagocytosis of cholesterol crystals (CCS) or calcium phosphate crystals and intracellular crystals formed by ox-LDL led to lysosomal instability and leakage of lysosomal enzyme cathepsin. Cathepsin B combines with the C-terminal LRRs domain of NLRP3 inflammasome to degrade inhibitors of NLRP3 inflammasome, accelerate the recruitment of ASC and pro-caspase-1, and activate NLRP3 inflammasome (14, 26).

#### 3.2.3. Mitochondrial dysfunction

Mitochondrial dysfunction and the release of mitochondrial ROS (mtROS) and mitochondrial DNA (mtDNA) into cytoplasm are key upstream signals for NLRP3 activation (19). Mitochondria are the main site of cell energy metabolism and a major production source of ROS in cells. A large number of ROS (mtROS) can be produced in oxidative phosphorylation and cellular stress. MtROS and Ca^2+^ open mitochondrial channels, known as mitochondrial permeability transition protein (MPT). Studies have shown that the amount of mtDNA released into the cytoplasm depends on the pore size of MPT and the level of mtROS. MtDNA released into the cytoplasm is oxidized as oxidized mitochondrial DNA (ox-mtDNA) and activates NLRP3 inflammasome. Immunoprecipitation showed that ox-mtDNA was consistent with NLRP3 (27).

#### 3.2.4. Oxidative stress

Studies have shown that ATP, lipopolysaccharide (LPS) and cholesterol crystals can activate NLRP3 inflammasome by promoting ROS. Disproportionate ROS levels promote activation of NLRP3 inflammasome and initiate gasdermin D (GSDMD)-mediated pyroptosis (28).

While nuclear factor-E2-related factor 2 (Nrf2) can partly inhibit NLRP3 activation in oxidative stress. On the one hand, Nrf2 inhibits NLRP3 activation by regulating the basic level of antioxidant genes and limiting the level of ROS after induction. By contrast, Nrf2 source gene transcription decreased NF-κB activates and down regulates inflammasome components NLRP3, caspase-1 and IL-1β (27) while restricting IL-18 and IL-3 expression.

## 4. The activation and regulation of ALDH2 in AS

Mitochondrial aldehyde dehydrogenase 2 (ALDH2) is a major aldehyde metabolizing enzyme for acetaldehyde and other toxic aldehydes located in mitochondria. Some aldehydes with strong electrophilicity, high activity and toxicity, such as 4-hydroxy-2-nonenal (4-HNE) and malondialdehyde (MDA), play an important role in the development of AS (29, 30). Accumulation of toxic aldehydes promotes oxidative stress, impaired calcium binding, damage to the endothelium, vasoconstriction, and thrombosis (31) which result in cardiovascular diseases. ALDH2 may have beneficial cardiovascular outcomes for cardiac hypertrophy, heart failure, myocardial I/R injury, reperfusion, arrhythmia, coronary heart disease and atherosclerosis (32).

Acetaldehyde is oxidized to acetate prior to elimination to the blood by ALDH2. The Glu487Lys polymorphism at codon 487 in the ALDH2 gene causes substitution of glutamate (Glu) by lysine (Lys). This polymorphism exists in approximately 40% East Asian populations (29). Such a polymorphism disrupts ALDH2 activity. Heterozygotes with ALDH2 mutation can reduce enzyme activity by 60–80%. The enzyme activity of homozygous mutant (AA) is only 1 to 4% of that of wild homozygous (GG) (33).

### 4.1. Aldehyde dehydrogenase 2 metabolizes toxic aldehydes and reduces oxidative stress

**Figure 2:**
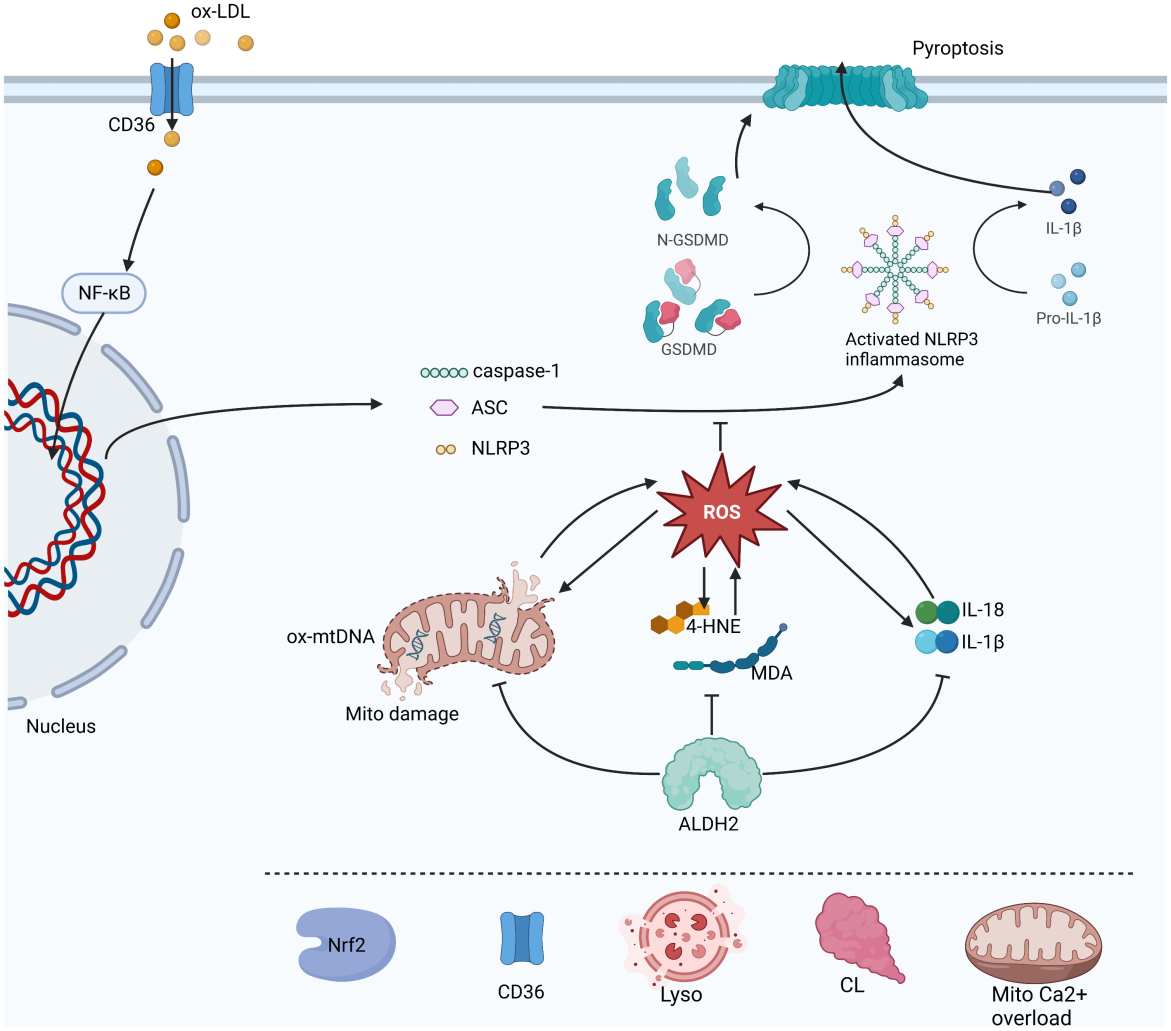
This is a figure of the process of NLRP3 inflammasome initiation and activation and the three pathways through which ALDH2 affects its activation are illustrated. ROS in direct inhibition is a common downstream of oxidative stress, inflammatory response and mitochondrial damage. In turn ROS also promotes these three aspects which are reflected by 4-HNE and MDA, mtDNA and IL-1β and IL-18 levels. ALDH2 can protect mitochondria, metabolize toxic aldehydes and reduce the production of inflammatory factors. Other possible factors involved in the regulation of NLRP3 by ALDH2 include Nrf2, lysosomal instability, cardiolipin (CL), mitochondrion Ca^2+^ overload and CD36.The sharp arrow indicates promotion, and the horizontal bar indicates inhibition.

The of aldehydes is due to the formation of adducts produced with biological macromolecules, such as protein or DNA, which interfere with their original physiological functions, and lead to inactivation or damage. In proteins, Michael additions from targeted thiol or amino groups come from the C3 of C2 = C3 double bond of 4-HNE. Schiff bases are formed from the reaction of primary amines of the C1 carbonyl groups of 4-HNE. Exocyclic adducts are also formed from the Michael addition of a C = C double bond of 4-HNE to the NH2-group of deoxyguanosine. These can directly damage the macromolecules and impair cellular functions (30). There is a vicious circle in which lipid peroxidation produces 4-HNE and causes accumulation of 4-HNE and formation adducts, which then binds to mitochondrial targets and cause damage to mitochondria, resulting in higher levels of ROS production (34, 35). ALDH2 has been shown to be involved in the elimination of these toxic endogenous aldehydes, decomposing them into non-toxic carboxylic acids inhibiting the creation of ROS and reduce oxidative stress (36) (Figure 2).

### 4.2. Aldehyde dehydrogenase 2 deficiency exacerbates DNA damage and oxidized mtDNA formation

Seo et al. (29) have shown that Aldh2-deficient hepatocytes create elevated levels of oxidized mtDNA. Results from *in vivo* and *in vitro* mechanistic studies revealed that Aldh2-deficient hepatocytes produce harmful oxidized mtDNA *via* extracellular vehicles (EVs) after CCl4 plus ethanol exposure. ALDH2 deficiency leads to superfluous accumulation of acetaldehyde and oxidative stress during the consumption of alcohol, which leads to mitochondrial DNA damage and base modifications potentially causing mitochondrial dysfunction.

N-(1,3-benzodioxol-5-ylmethyl)-2,6-dichloro-benza-mide (Alda-1) has been shown to be an activator of ALDH2. Daidzin is an antagonist of ALDH2 and cancels the effect of Alda-1. Alda-1 reverses the buildup of 4HNE-protein adducts and carbonylation in hearts undergoing heart failure. This preservation of cardiac function by Alda-1 is correlated with lowered levels of 4-HNE protein adducts and acetaldehyde and improved mitochondrial metabolism including a better oxidation–reduction status during severe myocardial infarction. For example, activation of ALDH2 lowered mitochondrial Ca^2+^-induced permeability and cytochrome C release in failing hearts. When examined in whole organs and organisms, ALDH2 activation by Alda-1 also protects against stress from aldehydes (37). Alda-1 counters the adverse effect of aldehyde load in the formation and development of chronic cardiac diseases leading to heart failure, such as myocardial infarction. It should be noted that alda-1 and daidzein alone had no effect on cell viability.

### 4.3. Oxidized low-density lipoprotein treatment impaired ALDH2 activity without changing its protein expression

Oxidized low-density lipoprotein treatment decreased ALDH2 protein activity without changing expression. Ox-LDL had no clear effects on ALDH2 protein levels. Interestingly, ox-LDL reduces ALDH2 activity compared to controls (38).

### 4.4. Nrf2 (nuclear factor erythroid 2(NF-E2)-related factor 2)

Research shows knockdown of Nrf2 canceled the 6-(methylsulfinyl) hexyl iso-thiocyanate (6-MSITC)-induced ALDH2 expression, suggesting that Nrf2 regulated the expression of ALDH2. Also, siRNA for Nrf2 canceled the induction of ALDH2 (39). Another study showed Nrf2 induced by Vitamin D could transcriptionally upregulate ALDH2 expression in order to support alcohol metabolism (40).

## 5. The relationship between ALDH2 and NLRP3 inflammasome

At present, the research on the relationship between ALDH2 and NLRP3 is still in its early stage. A total of six highly relevant articles were screened out by searching the keywords ALDH2 and NLRP3 (Table 1).

**Table 1:** This table compared six articles which were related to the relationship between NLRP3 and Aldehyde dehydrogenase 2 (ALDH2).

Article	Year	Research model	Experimental methods	Conclusions	Innovations/limitations
Zhenzhen Cao	2022	Septic lung injury model in mice by CLP method	Pulmonary ultrastructural observation: TEM TBA:MDA DHE fluorescence probe:ROS WB:ALDH2, Gpx4, Ptgs2, 4-HNE, NLRP3, Caspase-1, GSDMD, IL-1β, IL-18 and β-Actin	The activation of ALDH2 inhibits the initiation and activation of NLRP3 inflammasome by reducing oxidative stress	Interactions among ALDH2, pyroptosis, and ferroptosis may also occur
Wenlian Wang	2021	Diabetes mice, low dose ethanol	WB:ALDH2, NLRP3, ASC and caspase-1 fasting blood glucose (FBG), body weight and the lung/body coefficient; HE staining and Masson staining:lung histological changes	Low-dose ethanol increased ALDH2 protein expression and alleviated diabetes-induced lung injury by inhibiting the production of NLRP3 inflammasome	They did not continue the intensive investigation of the mechanisms related to the protection by low-dose ethanol
Ruiping Cao	2019	H9c2 cells, high glucose induced construction of myocardial cell line with high expression of mitochondrial ALDH2	CCK-8 Method:Cell Viability Mitosox Staining Method:Mitochondrial Oxidative Stress Mitochondrial ALDH2 Activity:mitochondrial ALDH2 activity assay kit RT-PCR:ALDH2 mRNA; ELISA:4-HNE, IL-1β, IL-18; WB:ALDH2, NLRP3, ASC, Caspase-1, IL-18, Caspase-3	ALDH2 overexpression alleviated high glucose-induced H9C2 cell pyroptosis through inhibiting inflammation and mitochondria-derived ROS release; overexpression of ALDH2 can inhibit the occurrence of apoptosis and also inhibit IL-18 expression	They transfected H9C2 cardiac cells with the lentivirus ALDH2 gene *in vitro*
Pinfang Kang	2019	Rat cardiomyocyte, dai dzin, Alda-1	MTT assay:cell viability and ATP content 450 nm absorbance:ALDH2 activity DHE staining:Superoxide production method ELISA:TNF-α, IL-6 and IL-1β; qPCR:ALDH2, Col I, Col III, RIP1, RIP3 and MLKL; WB:ALDH2, RIP1, RIP3, MLKL, NLRP3, ASC, MMP14和TIMP4; flow cytometry:Apoptosis and necroptosis measurement	Increasing ALDH2 expression can improve myocardial fibrosis induced by high glucose intervention through inhibiting the occurrence of necroptosis and inflammation reaction. ALDH2 regulates ROS release and inhibits inflammatory response	Daidzein was added to inhibit ALDH2 to verify the effect of ALDH2
Youshun Xu	2020	RAW264.7 cells, ox-LDL stimulation	ALDH2 activity assays PCR and WB:NLRP3, pro-caspase-1, caspase-1, pro-IL-1β, IL-1β ROS Assay Kit:ROS ELISA:IL-1β TUNEL method:DNA damage	Alda-1 alleviates ox LDL induced NLRP3 inflammasome activation by reducing oxidative stress; ox LDL weakened the activity of ALDH2 without changing its protein expression	It is involved in the initiation and activation of NLRP3 and its downstream; The mechanism research is more detailed
Mengyuan Diao	2020	Pig model after cardiac arrest and resuscitation, Alda-1, myocardium, hippocampus and cortex	ELISA:cardiac troponin I, neuron-specific enolase, L-1β, IL-18, 4-HNE WB:NLRP3, ASC, Caspase-1, GSDMD and ALDH2 Immunofluorescence Staining:Cleaved Caspase-1 and GSDMD expression in myocardium, hippocampus and cortex thiobarbituric acid:Lipid peroxidation(MDA Level) ALDH2 Activity Assay kit: ALDH2 activity	The activation of ALDH2 by Alda-1 promotes the clearance of MDA and 4-HNE and the suppression of ROS generation, then inhibits NLRP3 inflammasome activation and its downstream Caspase-1 cleavage, subsequently reduced membrane pore formation and proinflammatory cytokines production, and eventually inhibited cell pyroptosis	Multiple organizations

Blue represents cell experiments and yellow represents animal experiments.

### 5.1. The confirmed influence pathway of ALDH2 on NLRP3

Reactive oxygen species are reactive chemicals containing oxygen such as peroxides, superoxides, hydroxyl radicals, singlet oxygen, etc., that play an important role innate immunity and the cardiovascular system (41). ROS activates NLRP3 inflammasome as a common downstream pathway from different stimuli (42). In addition, ROS caused damage to DNA, damage to the permeability of the mitochondrial membrane and aggravate inflammation, ultimately leading to cell pyroptosis (42) (Figure 2).

Aldehyde dehydrogenase 2 has various functions, including reducing the production of oxygen free radicals, consumes aldehydes and 4-HNE, and protecting mitochondrial function (43, 44). It may affect the common upstream ROS activated by NLRP3 from the following three aspects.

### 5.2. Aldehyde dehydrogenase 2 reduces oxidative stress to alleviated NLRP3 initiation and activation

In the endothelium of atherosclerosis, lipid peroxidation produces toxic metabolites such as 4-HNE and MDA. These aldehydes are usually used as markers of oxidative stress because of their stable structure. As previously mentioned, these aldehydes can diffuse into and out of cells and attack target molecules, expand cell damage and aggravate oxidative stress, thereby producing more ROS. ROS in turn attacks or reacts with lipids, aggravating lipid peroxidation and allowing damage to continue.

Enzyme deficiency of ALDH2*2 is linked with elevated susceptibility to oxidative stress due to the accumulation of toxic aldehydes (45). Malondialdehyde (MDA), reactive oxygen species (ROS) and 4-hydroxy-2-nonenal (4-HNE) can reflect the level of oxidative stress. Chen et al. and Zhang et al. (37, 46) have confirmed that ALDH2 in addition to decomposing 4-HNE and MDA, can also reduce 4-HNE protein adduct formation, lessening the severity of oxidative damage by acetaldehyde and its metabolites (47). In addition, ALDH2 activation by Alda-1 is partially resolved by lessening of the production of ROS and extend the slowing or prevention of NLRP3 inflammasome-induced pyroptosis (36).

**Figure 3:**
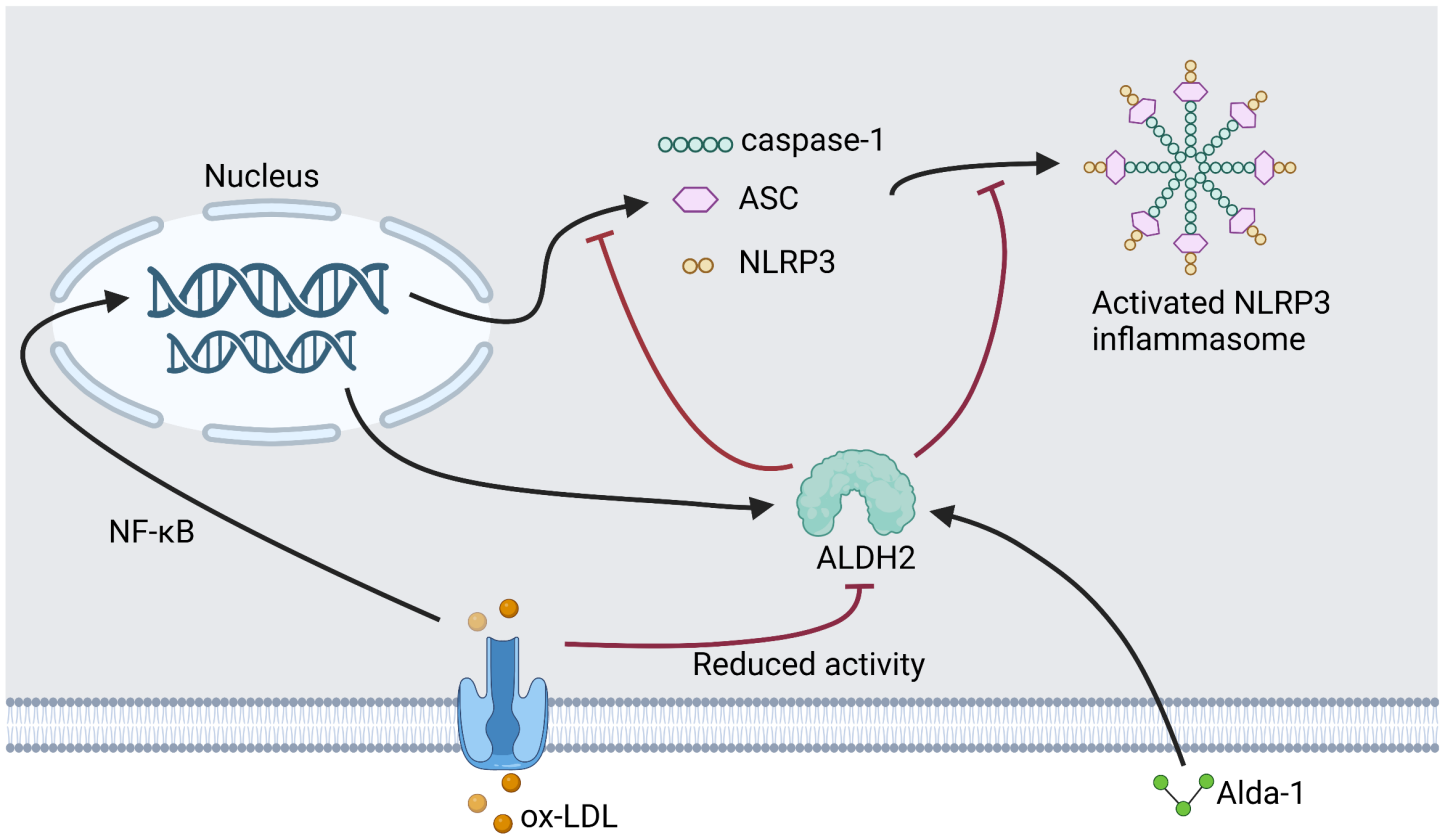
This figure shows our previous research plan and results. Add Alda to ox-LDL treated RAW264.7.

In our previous study, we assayed RAW264.7 for oxidative stress after treatment with ox-LDL and Alda-1. We demonstrated that ox-LDL elevated the generation of ROS, while treatment with Alda-1 decreased ROS significantly. Administration of Alda-1 made elevated 4-HNE expression close to baseline levels. Alda-1 could inhibit two phases (initiation and activation) of ox-LDL-mediated NLRP3 inflammasome activation. Cell pyroptosis was also relieved due to the inhibition of caspase-1 activation. Mechanically, we found that Alda-1-inhibited NLRP3 activation induced by ox-LDL was mediated by reducing oxidative stress. Taken together, ALDH2 activation inhibited initiation and activation of NLRP3 inflammasome *via* reducing oxidative stress (38) (Figure 3).

In another study, the expression of cleaved Caspase-1 and GSDMD were reduced in myocardium through immunofluorescence staining Inhibiting the production of ROS could also inhibit the cleavage of Caspase-1, reducing the formation of pores in the membrane and the production of proinflammatory cytokines, and finally inhibit the pyroptosis of cells (36). The protective effect of ALDH2 was abolished with the addition of daidzin, which blocks the ALDH2 activation role of Alda-1 (42). Lastly, pro-inflammatory cells may release ROS and further aggravate oxidative stress (41).

### 5.3. Aldehyde dehydrogenase 2 reduces inflammatory reaction to alleviated NLRP3 initiation and activation

When endothelial cells(ECs)are activated in AS, inflammatory factors are expressed that attract lymphocytes and monocytes that bind to the endothelium and infiltrate the arterial wall. Cells derived from innate immune system, such as macrophages, create ROS such as superoxide (O2^–^) and hydrogen peroxide (H2O2), aimed to destroy pathogens. ROS release increases in inflammatory conditions and chronic inflammation can cause oxidative stress which leads further to endothelial dysfunction and trigger excessive more production of ROS (41).

ROS activates Transcription factors such as nuclear factor (erythroid-Derived 2)-like 2 (NFE2L2), NF-κB and Activator Protein 1 (AP-I). These factors regulate the gene expression of adhesion molecules and chemokines causing buildup of macrophages and granulates, inflammatory that further release ROS and aggravate current oxidative stress (41).

In addition to increasing oxidative damage, Excessive ROS further promotes production of pro-inflammatory factors including IL-1β and tumor necrosis factor α (TNF-α) in addition to lipid peroxidation products, such as MDA and 4-HNE that cause severe damage (28). Damaged cells also release contents, further inducing an inflammatory reaction creating the accumulation of ROS that could cascade into production of more pro-inflammatory factors (48).

As mentioned earlier, ROS can directly activate NLRP3 inflammasome which also activates Caspase-1. Caspase-1 cleaves the cytokines pro-IL-1β and pro-IL-18 into IL-1β and IL-18 and also cleaves GSDMD, which leads to the creation of pores in the plasma membrane, causing the release of IL-1β and IL-18 from the cells and further expand inflammation (27, 36).

In Diao et al.’ s study (36), treatment with ALDH2 activator (Alda-1) in swine post cardiac arrest show significantly improved recovery in the heart, and improved oxidative stress and inflammatory responses after resuscitation from the inhibition of ROS-mediated NLRP3 inflammasome activation, decreasing pyroptosis mediated by GSDMD.

In a CLP-induced sepsis lung injury model, expression of inflammasome proteins NLRP3, caspase-1 and pyroptosis GSDMD release inflammatory cytokines IL-1β and IL-18. ALDH2 activation with Alda-1 alleviated lung injury, and the expression of NLRP3, Caspase-1, GSDMD, IL-1β, and IL-18 was reduced (28).

In another experiment, RAW264.7 murine monocyte/macrophage treated with human ox-LDL with or without ALDH2 agonist Alda-1 showed that ox-LDL induced NLRP3 and pro-IL-1β transcription was significantly reduced by Alda-1. As detected by immunoblotting, Alda-1 also attenuated protein levels of NLRP3 and pro-IL-1β (38). ALDH2 inhibits activation of NLRP3 inflammasome expression of IL-18 in impaired myocardial cells induced by high glucose (49). At the same time, IL-1β is also the end product of NLRP3 inflammasome activation and expand the inflammatory response and bind to IL-1R of macrophages, which promotes initiation of NLRP3 (50).

### 5.4. Aldehyde dehydrogenase 2 reduces mitochondrial damage to alleviated NLRP3 initiation and activation

Damage-associated molecular patterns(DAMP), such as damaged mitochondrial DNA, activates the NLRP3 inflammasome (51). Due to their oxidative reactions, Mitochondria are the major sources of ROS (52). Higher ROS levels may lead to a ROS burst leading to destruction of mitochondria by prolonging mitochondrial permeability transition pore (mPTP) openings (53). Thus, compared to other organelles, mitochondria are more susceptible to oxidative injury (54). Several studies confirmed that mitochondrial ROS (mtROS)is required for activation of the NLRP3 inflammasome (55, 56). Cellular stress produces a large amount of mtROS, while 4-HNE is an important marker of oxidative stress. Excessive 4-HNE adduct formation impairs mitochondrial metabolism, protein quality, and contractility (36).

Deficiency in ALDH2 results in an increased permeability in the mitochondria, altered mitochondrial membrane potential, increased damage to DNA, and reduced oxidative phosphorylation (45). Mitochondrial Oxidative Stress Measurement by Mitosox suggest that mitochondrial oxidative stress increased in high-glucose(HG)treatment. H9C2 cardiac cells transfected with the ALDH2 gene and reporter show fluorescence intensity in cells overexpressing the ALDH2 gene was significantly weakened compared with HG and HG + GFP, indicating the decrease of mitochondrial oxidative stress (49).

In a rat model of CA and resuscitation, the enhancement of ALDH2 expression attenuated post-resuscitation myocardial dysfunction. ALDH2 inhibited mitochondria ROS (mROS) triggered by ox-LDL treatment. Zhu et al. (57) show that ALDH2 overexpression inhibits mROS induced production by ox-LDL treatment.

### 5.5. Reasonably speculate the influence pathway of ALDH2 on NLRP3

#### 5.5.1. Nuclear factor-E2-related factor 2

Nuclear factor-E2-related factor 2 is a regulator for antioxidant proteins that protect against oxidative damage by regulating the basic level of induced expression of antioxidant genes to maintain cell survival during oxidative stress. In normal conditions, Nrf2 binds to Kelch-like ECH-associated protein 1 (Keap1) ubiquitylating Nrf2 leading to degradation. In conditions of increased free radicals, Nrf2 dissociates from Keap1 and trans-locates to the nucleus from the cytoplasm, and binds to the antioxidant response element, which enables transcription of antioxidant genes, which lead to resistance to oxidative stress and inflammation.

Nuclear factor-E2-related factor 2 inhibits NLRP3 activation by limiting ROS levels. Nrf2 source gene transcription attenuates NF-κB, activates and down regulates inflammasome components NLRP3, caspase-1, IL-1β and IL-18, thereby limiting NLRP3 inflammasome activity (27).

Nrf2 is an important downstream target of Sirt1 signaling. Nrf2 and Sirt1 have anti-oxidative stress functions when stimulated by free radicals. Previous studies have found that Nrf2 and Sirt1 were detected in Alda1-treated diabetic rats model group by western blot method (48, 58).

Melatonin has been experimentally proven to promote translocation of Nrf2 into the nucleus and increase expression of target anti-oxidative genes. Conversely, knocking down Nrf2 increases IL-1β andNLRP3 mRNA while diminishing melatonin’s protective capacity against the production of mitochondrial ROS. In addition, some studies suggest that melatonin inhibit NLRP3 inflammasome by suppressing ROS production (58). Another study showed that gallic acid reduced the production of mtROS by up regulating the expression of Nrf2, thereby inhibiting the activation of NLRP3 inflammasome (59).

6-(Methylsulfinyl) hexyl iso-thiocyanate (6-MSITC) has anti-oxidative, anti-inflammatory, and anti-cancer effects. In Kitakaze et al.’s study, 6-MSITC induced mitochondrial ALDH2 through the Nrf2 pathway and nuclear translocation of Nrf2. Knockdown of Nrf2 negated expression of 6-MSITC-induced ALDH2, suggesting that Nrf2 regulated the expression of ALDH2 (39).

In a study of liver fibrosis, liver fibrosis can be significantly attenuated by exogenous administration of Alda-1 by up-regulating the antioxidant Nrf2/HO-1 pathway and activating Parkin-related mitophagy (60). While Chen et al.’s study discovered a novel role of Nrf2/HO-1 signaling in NLRP3 production, Chen et al. also showed activation of the NLRP3 inflammasome can be activated by Nrf2/HO-1 signaling (61).

In addition, ALDH2 overexpression enhances ERK phosphorylation and upregulated Nrf2 transcription *via* a newly identified TRE in the exon 1 of Nrf2 (40).

#### 5.5.2. CD36

Macrophages become foam cells through CD36 uptake and accumulation of ox-LDL in atherosclerosis. The accumulation of foam cells contributes to plaque lipid storage and plaque growth. Transcription factors of the NF-κB family participate in many key physiological responses, such as the inflammatory response (62) and NF-kB signaling is active upstream of NLRP3 inflammasome (21, 63). After ox-LDL enters the cells, it upregulates the expression of NLRP3 inflammasome related protein through NF-κB pathway and promotes its initiation.

Studies have shown the effect of ALDH2 on CD36 in the formation of foam cells. Wei et al. show that the expression of CD36 was decreased in ALDH2-deficient macrophages while ox-LDL uptake is primarily mediated by CD36. This suggests that ALDH2 may reduce the ox-LDL uptake of macrophages by reducing the expression of CD36, and then inhibit activation of NF-κB and expression of NLRP3 (64).

#### 5.5.3. Lysosomes

Phospho-mALDH2 translocated to the nucleus in HDAC3-suppresses the transcription of the lysosomal H^+^ pump subunit of ATP6V0E2, critical for maintaining lysosomal function, autophagy, and degradation of oxidized low-density lipid protein. The loss of this critical pathway accelerates lysosomal dysfunction, mediating enhanced foam cell formation (57, 65).

#### 5.5.4. Cardiolipin (CL)

There is evidence that mitochondria are the docking site of NLRP3 inflammasome assembly. In normal state, NLRP3 is a cytoplasmic protein related to endoplasmic reticulum (ER), but when activated, NLRP3 will be associated with mitochondria and mitochondrial related membrane proteins, especially with cardiolipin (CL). Cardiolipin is a phospholipid in the inner membrane of mitochondria, which can bind to the leucine rich LRR domain in NLRP3. When mitochondrial oxidative stress occurs, CL will be exposed to the outer membrane and bind to NLRP3 and caspase-1 independently as binding sites. The interaction between the three is necessary for the assembly and activation of NLRP3 inflammasome (27).

While ALDH2 is located in mitochondria, which can effectively reduce mitochondrial oxidative stress and protect mitochondria from damage, it is speculated that ALDH2 may hinder NLRP3 inflammasome by reducing CL externalization.

#### 5.5.5. Mitochondrial Ca^2+^ overload

Mitochondria have high calcium buffering capacity and are important organelles for regulating intracellular calcium concentration. The opening of intracellular Ca^2+^ channels and the release of endoplasmic reticulum Ca^2+^ not only directly promote the activation of NLRP3 (24), but also increase of intracellular Ca^2+^ concentration, leading to the increase of mitochondrial Ca^2+^. Mitochondrial Ca^2+^ overload stimulates the production of ROS and stimulates mitochondrial permeability transition pore (mPTP) opening and mitochondrial growth, ending with mitochondrial damage (66). ALDH2 activation decreases mitochondrial Ca^2+^-induced permeability in failing hearts (37). Mochly-Rosen et al.’s (67) suggest that ALDH2 is a downstream actor of protein kinase C type ε (εPKC) and mPTP opening is just one target of εPKC. Other studies have shown that activating ALDH2 can inhibits MPTP opening (68). Whether ALDH2 can also play such a role in macrophages in atherosclerosis remains to be explored.

## 6. Conclusion

Many researchers have explained the relationships and potential functions of ALDH2 and NLRP3 inflammasome in ASCVD. ALDH2 is an important enzyme of toxic aldehyde metabolism located in mitochondria that plays a major role in the metabolism of toxic aldehydes such as 4-HNE and MDA. ROS is a common downstream pathway for NLRP3 inflammasome (69). ALDH2 can reduce the multiple sources of ROS, such as oxidative stress, inflammation, and mitochondrial damage, thereby reducing the activation of NLRP3 inflammasome. On the other hand, according to the downstream of ALDH2 and the upstream of NLRP3, the molecules and related mechanisms of ALDH2 on NLRP3 inflammasome are comprehensively expounded. The potential mechanism may provide new inroads for the treatment of ASCVD.

## Author contributions

X-yS wrote the paper. R-jL revised the manuscript. All authors contributed to the article and approved the submitted version.

## Funding

This study was supported by Shandong Province Natural Science Foundation (ZR2021MH405).

## Conflict of interest

The authors declare that the research was conducted in the absence of any commercial or financial relationships that could be construed as a potential conflict of interest.

## Publisher’s note

All claims expressed in this article are solely those of the authors and do not necessarily represent those of their affiliated organizations, or those of the publisher, the editors and the reviewers. Any product that may be evaluated in this article, or claim that may be made by its manufacturer, is not guaranteed or endorsed by the publisher.
